# Low-dose vs. standard-dose intravenous alteplase for acute ischemic stroke with unknown time of onset

**DOI:** 10.3389/fneur.2023.1165237

**Published:** 2023-04-28

**Authors:** Zekun Wang, Kangxiang Ji, Qi Fang

**Affiliations:** Department of Neurology, The First Affiliated Hospital of Soochow University, Suzhou, China

**Keywords:** acute ischemic stroke, alteplase, dose, intravenous thrombolysis, extended time window, wake-up stroke

## Abstract

**Background:**

Standard-dose intravenous alteplase for acute ischemic stroke (AIS) in the unknown or extended time window beyond 4.5 h after symptom onset is both effective and safe for certain patients who were selected based on multimodal neuroimaging. However, uncertainty exists regarding the potential benefit of using low-dose alteplase among the Asian population outside the 4.5-h time window.

**Methods:**

Consecutive AIS patients who received intravenous alteplase between 4.5 and 9 h after symptom onset or with an unknown time of onset guided by multimodal computed tomography (CT) imaging were identified from our prospectively maintained database. The primary outcome was excellent functional recovery, defined as having a modified Rankin scale (mRS) score of 0–1 at 90 days. Secondary outcomes included functional independence (an mRS score of 0–2 at 90 days), early major neurologic improvement (ENI), early neurologic deterioration (END), any intracranial hemorrhage (ICH), symptomatic ICH (sICH), and 90-day mortality. Propensity score matching (PSM) and multivariable logistic regression models were used to adjust for confounding factors and compare the clinical outcomes between the low- and standard-dose groups.

**Results:**

From June 2019 to June 2022, a total of 206 patients were included in the final analysis, of which 143 were treated with low-dose alteplase and 63 were treated with standard-dose alteplase. After accounting for confounding factors, we observed that there were no statistically significant differences between the standard- and low-dose groups with respect to excellent functional recovery [adjusted odds ratio = 1.22 (aOR), 95% confidence interval (CI): 0.62–2.39; adjusted rate difference (aRD) = 4.6%, and 95% CI: −11.2 to 20.3%]. Patients of both groups had similar rates of functional independence, ENI, END, any ICH, sICH, and 90-day mortality. In the subgroup analysis, patients aged ≥70 years were more likely to achieve excellent functional recovery when receiving standard-dose rather than low-dose alteplase.

**Conclusion:**

The effectiveness of low-dose alteplase might be comparable to that of standard-dose alteplase in AIS patients aged <70 years with favorable perfusion-imaging profiles in the unknown or extended time window but not in those aged ≥70 years. Furthermore, low-dose alteplase did not significantly reduce the risk of sICH compared to standard-dose alteplase.

## Introduction

Intravenous thrombolysis (IVT) is currently the standard of care for patients with acute ischemic stroke (AIS) who present within 4.5 h of symptom onset or the last known well time (1). However, the overall rates of IVT for AIS remain low, with only 7.3% in Europe and 5.64% in China (2, 3). One of the main reasons for withholding thrombolytic therapy is the late time window of presentation of patients. The Extending the time for Thrombolysis in Emergency Neurological Deficits (EXTEND) trial showed that, by using CT or MRI perfusion imaging to identify potentially salvageable penumbra, IVT was still safe and effective for AIS beyond the 4.5-h time window (4). This finding was further supported by the meta-analyses of the EXTEND, the European Cooperative Acute Stroke Study-4 (ECASS-4) (5), and the Echoplanar Imaging Thrombolytic Evaluation Trial (EPITHET) (6–8). Accordingly, the 2021 European Stroke Organization (ESO) guidelines recommend IVT for AIS patients upon awakening from sleep, who have CT or MRI core/perfusion mismatch within 9 h from the midpoint of sleep and for whom mechanical thrombectomy is either not indicated or not planned (quality of evidence: moderate; strength of recommendation: strong) (9). The ESO guidelines also advocate the EXTEND protocol used in IVT for patients with ischemic stroke within the 4.5–9 h duration (quality of evidence: low; strength of recommendation: strong) (9).

It has been reported that Asians are at a higher risk for symptomatic intracranial hemorrhage (sICH) following IVT with standard-dose (0.9 mg/kg) alteplase compared to other ethnic groups (10). The question of whether a lower dose of alteplase could improve the safety of thrombolytic therapy while maintaining its effectiveness has long been debated. The ENhanced Control of Hypertension ANd Thrombolysis strokE stuDy (ENCHANTED), as the only randomized controlled trial (RCT), demonstrated that low-dose alteplase (0.6 mg/kg) did not meet the non-inferiority criteria compared to standard-dose alteplase in terms of reducing death and disability, defined by the modified Rankin scale (mRS) scores of 2–6 at 90 days, within 4.5 h of stroke onset. Nevertheless, low-dose alteplase was non-inferior in overall functional recovery, as assessed through a shift analysis of 90-day mRS scores. There was a lower incidence of sICH reported in the low-dose alteplase group (11). Currently, the approved dosage of intravenous alteplase for AIS in Japan is 0.6 mg/kg, and low-dose alteplase is widely used in several other Asian countries owing to the anticipated lower risk of sICH and cost reduction (12–14).

The clinical trials published on IVT guided by perfusion imaging in the unknown or extended time window almost consistently utilized the standard-dose alteplase (4–6). Few studies have investigated the use of low-dose alteplase outside the 4.5-h time window (15). Given the concern for the risk of IVT-associated ICH in Asians, it remains unclear whether low-dose alteplase can achieve better safety and similar effectiveness as standard-dose alteplase beyond 4.5 h. Therefore, our study aimed to evaluate the effectiveness and safety of two different doses of alteplase for AIS patients with favorable perfusion imaging profiles within 4.5 and 9 h from symptom onset or with unknown time of onset, using real-world clinical data obtained from a high-volume tertiary stroke center in China.

## Methods

### Study design and patient selection

There are around 300 cases of IVT and 100 cases of endovascular thrombectomy at our tertiary stroke center every year. Since 2014, all patient profiles, including general information, laboratory test results, imaging results, neurological deficit scores at different stages, treatment modalities, and follow-up results, have been prospectively recorded in the stroke electronic database known as the MEDICAL SYSTEM. This cohort study retrospectively analyzed the data of consecutive AIS patients treated with intravenous alteplase in the unknown or extended time window beyond 4.5 h, between June 2019 and June 2022, by reviewing the database. The inclusion criteria were as follows: (1) patients receiving IVT with alteplase (a) between 4.5 and 9 h from symptom onset or (b) with unknown time of stroke onset and the last known well time of >4.5 h (including patients with symptoms on awakening [wake-up stroke] or unable to report the time of symptom onset) and onset-to-needle time of <9 h (the estimated time of stroke onset was defined by the midpoint of the last known well time and symptom recognition time); (2) age over 18 years; (3) excellent functional status before stroke onset (mRS score < 2); and (4) target perfusion–core mismatch, as determined by automated perfusion imaging (the mismatch ratio between perfusion-lesion volume and ischemic-core volume is >1.2, the absolute difference is greater than 10 ml, and the ischemic-core volume is less than 70 ml). The exclusion criteria were as follows: (1) a baseline National Institutes of Health Stroke Scale (NIHSS) score of <4; (2) unavailability of perfusion imaging results; and (3) incomplete individual patient data.

### Neuroimaging protocol and treatment

Our institutional algorithm for the management of AIS in the unknown or extended time window was established based on national and international guidelines (9, 13). Suspected AIS patients who entered our stroke green channel beyond 4.5 h from the last known well time would first undergo multimodal computed tomography (CT) scans, including non-contrast CT (NCCT), CT angiography (CTA), and CT perfusion (CTP). GE Revolution CT scanners (GE Healthcare, Ltd. Co, USA) were used for these examinations. The NCCT imaging was performed at 120 kV, 320 mAs, with a slice thickness of 0.625 mm and reconstructed slice thickness at 5 mm. The CTP parameters were set to 80 kV and 150 mAs. Continuous gantry rotations in the cine mode (every 2 s for 60.3) were implemented during the intravenous injection of 40 ml of ioversol (Ultravist 370; Bayer Healthcare, Berlin, Germany) at a rate of 5 ml/s, followed by the administration of 50 ml of saline. The CTA was acquired using an intravenous injection of 40 ml of ioversol at a flow rate of 5 ml/s, followed by 50 ml of saline. CTP imaging on all patients was done using the MIStar automatic software (Apollo Medical Imaging Technology, Melbourne, Australia) to quantitatively calculate the volume of perfusion lesion and ischemic core. As previously validated, MIStar defines the perfusion lesion as the tissue with a delay time (DT) of >3 s and the ischemic core as the tissue with cerebral blood flow of <30% (16). The mismatch ratio was assessed as the ratio of perfusion-lesion volume to that of the ischemic core.

After excluding ICH and massive cerebral infarction on baseline NCCT, patients who met the target mismatch criteria (mismatch ratio >1.2, absolute mismatch volume >10 ml, and ischemic core volume <70 ml) validated by the EXTEND trial and the additional eligibility criteria for IVT as recommended in the guidelines were treated with intravenous alteplase (4, 9, 13). In clinical practice, the selection of standard- or low-dose alteplase was determined by the treating physicians based on a comprehensive evaluation of the patients and professional discretion after taking into account the risk of bleeding. In general, high-risk factors for sICH after IVT include older age, higher baseline NIHSS, history of atrial fibrillation, elevated serum glucose upon admission, and so on (17, 18). Alteplase (Actilyse; Boehringer Ingelheim, Ingelheim, Germany) was administered intravenously as a 10% bolus and 90% infusion over 1 h for a standard dose of 0.9 mg/kg or as a 15% bolus and 85% infusion over 1 h for a low dose of 0.6 mg/kg. Follow-up imaging (CT or MRI) was performed 24 h after IVT to assess the presence of ICH or earlier in the case of neurologic deterioration.

### Analyzed parameters and outcome measures

The parameters analyzed for the enrolled patients were as follows: (1) demographic information [sex, age, weight, pre-stroke mRS score, systolic blood pressure (SBP), and diastolic blood pressure (DBP)]; (2) comorbidities [hypertension, diabetes mellitus, hyperlipidemia, atrial fibrillation, current smoking, previous stroke or transient ischemic attack (TIA), and antiplatelet/anticoagulant use prior to stroke onset]; (3) laboratory test results [admission glucose, platelet count, prothrombin time (PT), and international normalized ratio (INR)]; (4) clinical characteristics (stroke severity, confirmed extended time window, and wake-up stroke); (5) imaging features [large vessel occlusion (LVO), anterior or posterior circulation stroke (ACS or PCS), Alberta Stroke Program Early CT Score (ASPECTS), volume of perfusion lesion and ischemic core]; (6) treatment and time metrics [doses of intravenous alteplase, mechanical thrombectomy (MT), onset-to-door time (ODT), door-to-needle time (DNT), and onset-to-needle time (ONT)]; and (7) modified Trial of ORG 10172 in Acute Stroke Treatment (TOAST) classification and duration of hospitalization. Stroke severity was evaluated using the NIHSS scores measured at the time of admission, at 24 h after admission, and at 7 days (or at discharge if sooner) after admission. A 90-day follow-up was performed via telephone calls or outpatient visits to assess the mRS score.

The primary outcome of this study was the percentage of patients who achieved excellent functional recovery at 90 days after the index stroke event, as defined by an mRS score of 0–1. Secondary outcomes included the percentage of patients achieving functional independence at 90 days, as defined by an mRS score of 0–2, and the incidences of early major neurologic improvement (ENI), early neurologic deterioration (END), any ICH, sICH, and 90-day all-cause mortality following stroke onset. ENI was defined as a reduction in the NIHSS score by at least 8 points or a score of 0 or 1 at 24 h post-IVT. END was defined by an increase of four or more points in the NIHSS score, compared with baseline or the lowest NIHSS score, within 24 h of IVT. IVT-associated sICH was determined according to various criteria, including those outlined in the National Institute of Neurological Diseases and Stroke (NINDS) trial (19), European Cooperative Acute Stroke Study (ECASS) II (20), EACSS III (21), and Safe Implementation of Thrombolysis in Stroke Monitoring Study (SITS-MOST) (22).

### Statistical analysis

First of all, descriptive statistics were reported for baseline characteristics and outcomes in the two groups. Continuous variables with normal distribution were described as mean ± standard deviation (SD), while those with skewed distribution were described as median with interquartile range (IQR). Categorical variables were expressed in the form of frequency with proportions. Statistical differences between the two groups were analyzed using the chi-squared or the Fisher exact test for categorical variables, and the Mann–Whitney *U*-test or independent-sample *t*-test for continuous variables.

Second, we performed propensity score matching (PSM) to account for baseline imbalances between the standard- and low-dose groups. The propensity score was generated using a multivariable logistic regression model, with covariates including age, NIHSS, confirmed extended time window, perfusion lesion volume, posterior circulation stroke, onset-to-needle time, large vessel occlusion, and mechanical thrombectomy. Patients who received standard-dose therapy were matched at a ratio of 1:2 with those who received low-dose therapy based on the propensity score, without replacement, using the nearest neighbor matching and a caliper value of 0.15. Standardized mean differences (SMD) in covariate means were calculated after PSM to evaluate bias reduction. Regarding both primary and secondary outcomes, we calculated the odds ratios (OR) and absolute rate differences (aRD) with corresponding 95% confidence intervals (CI) in the PSM cohort. In addition, sensitivity analyses using multivariable logistic regression models were conducted to assess the robustness of the results by adjusting for covariates (age, sex, NIHSS, and variables with a *p*-value of <0.1 in the univariable analysis). Mortality and sICH were not adjusted due to their rarity.

Finally, to investigate which patients could benefit more from the current treatment (low- vs. standard-dose alteplase), we further explored the consistency of excellent functional recovery within the following subgroups: age (<70 vs. ≥70 years), NIHSS (<10 vs. ≥10), OTT (<360 vs. 360–540 min), confirmed extended time window (yes vs. no), LVO (yes vs. no), and MT (yes vs. no). A multiplicative term was entered into the binary logistic regression models to test for interaction.

All data analyses were conducted using the IBM SPSS Statistics software version 26.0 (IBM Corp., Armonk, NY, USA) and the MatchIt package of R software version 4.2.1. A two-tailed *p*-value of <0.05 was considered statistically significant.

## Results

### Baseline characteristics

**Figure 1:**
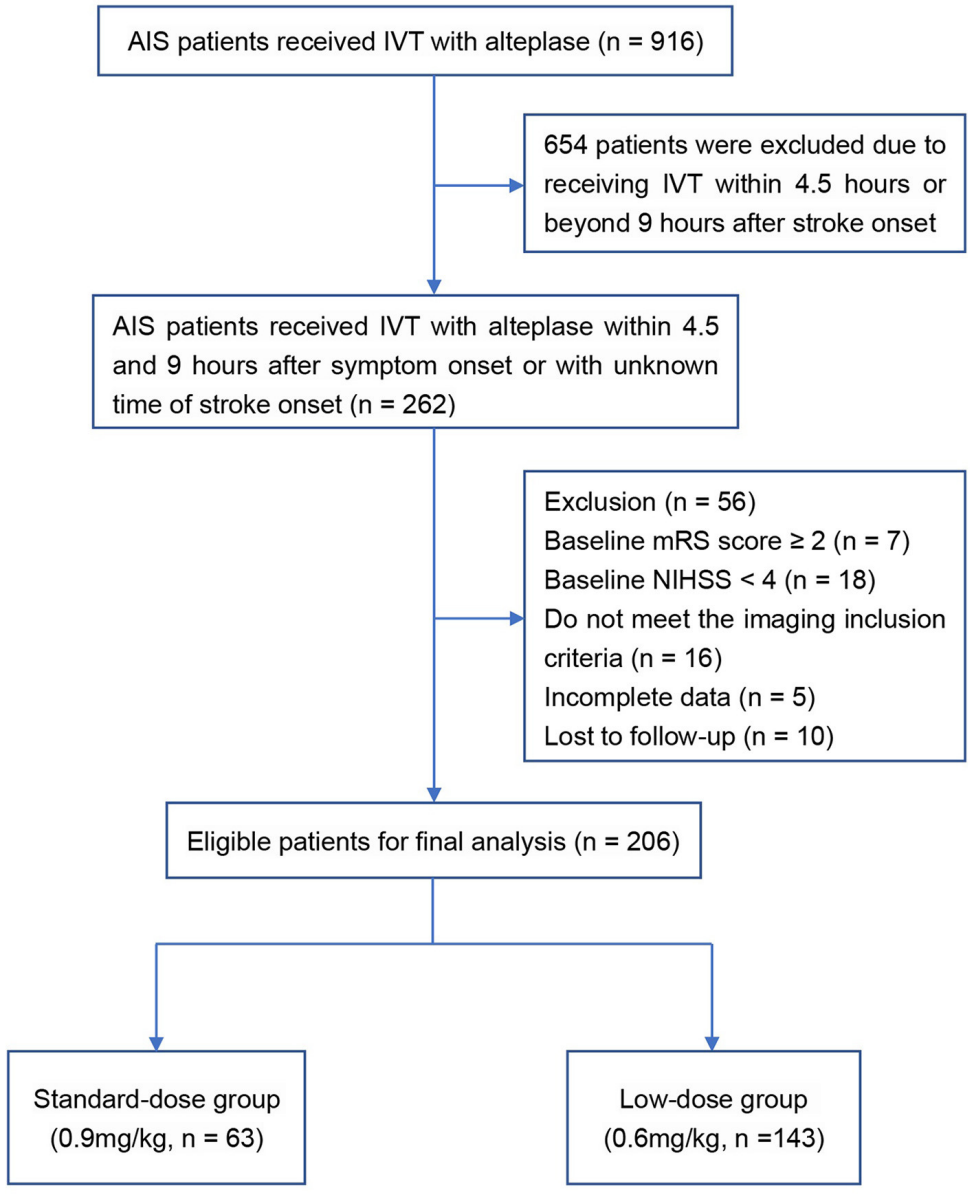
Flowchart of patient selection.

A total of 206 AIS patients who met the inclusion and exclusion criteria were enrolled in the final analysis (Figure 1). In the original cohort, 63 and 143 patients received standard- and low-dose intravenous alteplase within 4.5 to 9 h of symptom onset or within 9 h from the estimated time of stroke onset, respectively. Baseline demographics, medical history, and laboratory test results are summarized in Table 1, and the clinical and imaging profiles of patients are shown in Table 2. Patients in the low-dose group were significantly older than those in the standard-dose group [71 (58–80) vs. 67 (57–74) years, *p* = 0.017] and appeared to have a larger perfusion lesion volume [73 (33–129) vs. 52 (24–104), *p* = 0.053]. The proportion of patients in the confirmed extended time window beyond 4.5 h and the admission NIHSS scores were similar between the two groups. The ONT in the low-dose group was significantly longer than that in the standard-dose group [426 (372–503) vs. 416 (337–485) min, *p* = 0.043], whereas the DNT between the two groups was similar. After the 1:2 PSM, there were 59 and 93 patients in the standard- and low-dose groups, respectively. The overall baseline characteristics were balanced and comparable between the two groups in the PSM cohort (Tables 1, 2; Supplementary Figure 1).

**Table 1:** Baseline characteristics of patients treated with standard- vs. low-dose alteplase before and after PSM.

**Characteristics**	**Before PSM**			**After PSM**		
	**Standard dose (*****n*** = **63)**	**Low dose (*****n*** = **143)**	***p*** **-value**	**Standard dose (*****n*** = **59)**	**Low dose (*****n*** = **93)**	***p*** **-value**
Male sex, *n* (%)	43 (68.3)	96 (67.1)	0.874	40 (67.8)	67 (72.0)	0.576
Age (year)	67 (57–74)	71 (58–80)	0.017	67 (57–74)	67 (57–76)	0.856
Age ≥70 y, *n* (%)	26 (41.3)	77 (53.8)	0.096	24 (40.7)	38 (40.9)	0.982
Weight (kg)	68 (55–75)	67 (59–75)	0.861	68 (55–75)	68 (59–75)	0.491
SBP (mmHg)	154 (134–172)	155 (135–168)	0.373	153 (132–172)	151 (134–168)	0.267
DBP (mmHg)	87 (75–99)	88 (79–97)	0.870	87 (75–99)	86 (78–97)	0.923
**Medical history**						
Hypertension, *n* (%)	44 (69.8)	93 (65.0)	0.501	40 (67.8)	55 (59.1)	0.283
Diabetes mellitus, *n* (%)	17 (27.0)	42 (29.4)	0.727	17 (28.8)	27 (29.0)	0.977
Hyperlipidemia, *n* (%)	14 (22.2)	40 (28.0)	0.387	13 (22.0)	27 (29.0)	0.340
Atrial fibrillation, *n* (%)	13 (20.6)	39 (27.3)	0.312	13 (22.0)	22 (23.7)	0.817
Current smoker, *n* (%)	16 (25.4)	43 (30.1)	0.494	16 (27.1)	30 (32.3)	0.501
Previous stroke/TIA, *n* (%)	9 (14.3)	31 (21.7)	0.216	9 (15.3)	22 (23.7)	0.210
Prior antiplatelet use, *n* (%)	8 (12.7)	27 (18.9)	0.276	8 (13.6)	19 (20.4)	0.280
Prior anticoagulant use, *n* (%)	2 (3.2)	5 (3.5)	1.000	2 (3.4)	3 (3.2)	1.000
**Laboratory data**						
Glucose (mmol/L)	7.36 (6.00–10.01)	7.00 (6.10–8.83)	0.245	7.36 (5.95–10.01)	6.92 (5.92–8.48)	0.226
Platelet count (× 10^9^/L)	191 (170–231)	203 (168–243)	0.357	191 (162–231)	207 (170–245)	0.143
PT (s)	13.3 (12.7–13.9)	13.2 (12.6–13.9)	0.864	13.3 (12.7–14.0)	13.1 (12.7–13.8)	0.476
INR	1.03 ± 0.08	1.04 ± 0.09	0.635	1.03 ± 0.08	1.03 ± 0.09	0.917

PSM, propensity score matching; SBP, systolic blood pressure; DBP, diastolic blood pressure; TIA, transient ischemic attack; PT, prothrombin time; INR, international normalized ratio.

**Table 2:** Clinical and imaging characteristics of patients treated with standard- vs. low-dose alteplase before and after PSM.

**Characteristics**	**Before PSM**			**After PSM**		
	**Standard dose (*****n*** = **63)**	**Low dose** **(*****n*** = **143)**	***p-*** **value**	**Standard dose (*****n*** = **59)**	**Low dose (*****n*** = **93)**	***p*** **-value**
NIHSS score on admission	10 (6–15)	9 (6–14)	0.251	9 (6–14)	10 (7–16)	1.000
NIHSS score >15 on admission, *n* (%)	15 (23.8)	29 (20.3)	0.569	13 (22.0)	24 (25.8)	0.597
Wake-up stroke, *n* (%)	29 (46.0)	67 (46.9)	0.913	26 (44.1)	46 (49.5)	0.516
Confirmed extended time window, *n* (%)	17 (27.0)	50 (35.0)	0.260	17 (28.8)	31 (33.3)	0.559
**Time metrics**						
Stroke onset^a^ to door (min)	334 (249–404)	349 (291–414)	0.060	338 (253–406)	325 (273–404)	0.889
Stroke onset^a^ to needle (min)	416 (337–485)	426 (372–503)	0.043	419 (344–494)	403 (365–488)	0.727
Door to needle (min)	81 (72–90)	85 (72–100)	0.184	81 (72–90)	85 (71–99)	0.380
**Imaging result**						
ASPECTS^b^	8 (7–9)	8 (7–9)	0.948	8 (7–9)	8 (7–9)	0.945
Pc–ASPECTS^c^	9 (8–9)	9 (8–9)	0.831	9 (8–10)	9 (8–9)	0.589
Large–vessel occlusion^d^, *n* (%)	41 (65.1)	97 (67.8)	0.699	39 (66.1)	60 (64.5)	0.842
Volume of irreversibly injured ischemic–core tissue at initial imaging^e^ (mL)	4 (1–14)	4 (1–15)	0.992	4 (1–14)	4 (1–15)	0.876
Perfusion-lesion volume at initial imaging^f^ (mL)	52 (24–104)	73 (33–129)	0.053	52 (24–104)	66 (32–118)	0.368
Posterior circulation stroke, *n* (%)	15 (23.8)	20 (14.0)	0.084	13 (22.0)	16 (17.2)	0.460
**TOAST classification**, ***n*** **(%)**						
Large artery atherosclerosis	47 (74.6)	98 (68.5)	0.515	43 (72.9)	65 (69.9)	0.588
Cardioembolism	13 (20.6)	33 (23.1)		13 (22.0)	18 (19.4)	
Small artery occlusion	0 (0)	2 (1.4)		0 (0)	2 (2.2)	
Other determined etiology	1 (1.6)	8 (5.6)		1 (1.7)	6 (6.5)	
Undetermined etiology	2 (3.2)	2 (1.4)		2 (3.4)	2 (2.2)	
Mechanical thrombectomy, *n* (%)	18 (28.6)	36 (25.2)	0.610	16 (27.1)	25 (26.9)	0.974
Duration of hospitalization	11 (8–14)	11 (9–15)	0.377	12 (9–14)	11 (9–14)	0.974

PSM, propensity score matching; NIHSS, National Institutes of Health Stroke Scale; ASPECTS, Alberta Stroke Program Early CT Score; TOAST, modified Trial of ORG 10172 in Acute Stroke Treatment.

a Among patients with unknown time of onset, stroke onset is defined as the midpoint from last known well time to symptom recognition.

b ASPECTS score was available in 172 patients with anterior circulation ischemic stroke.

c Pc- ASPECTS score was available in 35 patients with posterior circulation ischemic stroke. One patient was diagnosed with concomitant anterior and posterior circulation stroke.

d Large-vessel occlusion is defined as occlusion of the internal carotid artery, first division and proximal portion of the second division of the middle cerebral artery, and vertebra-basilar artery.

e The volume of irreversibly injured ischemic-core tissue at initial imaging was calculated with the use of a threshold for cerebral blood flow of <30% of that in normal brain tissue.

f The perfusion-lesion volume was calculated as the volume of tissue with delay time exceeding 3 s.

### Clinical outcomes

The primary and secondary outcomes are presented in Table 3. Regarding the mRS scores of 0–1, 38.1% and 32.2% of patients achieved excellent functional recovery in the standard- and low-dose groups, respectively (OR = 1.30, 95% CI 0.70–2.41). For the mRS scores of 0–2, the corresponding proportions were 54.0% and 46.9% (OR 1.33, 95% CI 0.73–2.41). No significant differences were detected in the rates of ENI (25.4 vs. 18.9%, OR = 1.46, 95% CI 0.72–2.96), END (17.5 vs. 20.3%, OR = 0.83, 95% CI 0.39–1.79), and 90-day mortality (9.5% vs. 6.3%, OR = 1.57, 95% CI 0.53–4.61) between the standard- and low-dose groups. Patients treated with low-dose alteplase had lower odds of experiencing any ICH (10.5 vs. 19.4%, OR = 2.05, 95%CI 0.90–4.68) and sICH following different definitions compared to those treated with standard-dose alteplase, but the statistical differences were non-significant. One patient in the standard-dose group died prior to follow-up imaging, likely due to sudden cardiac arrest and circulatory failure.

**Table 3:** Primary and secondary outcomes of patients treated with standard- vs. low-dose alteplase before and after PSM.

**Clinical outcome**	**Standard dose (*n* = 63)**	**Low dose (*n* = 143)**	**Crude OR (95% CI)**	**Adjusted OR^a^ (95% CI)**	**Adjusted OR^b^ (95% CI)**	**Adjusted RD^b^ (95% CI)**
mRS 0–1 at 90 days, *n* (%)	24 (38.1)	46 (32.2)	1.30 (0.70–2.41)	1.28 (0.62–2.66)	1.22 (0.62–2.39)	4.6% (−11.2% to 20.3%)
mRS 0–2 at 90 days, *n* (%)	34 (54.0)	67 (46.9)	1.33 (0.73–2.41)	1.06 (0.52–2.19)	1.18 (0.62–2.27)	4.2% (−12.1% to 20.4%)
ENI, *n* (%)	16 (25.4)	27 (18.9)	1.46 (0.72–2.96)	1.25 (0.59–2.66)	1.33 (0.61–2.88)	5.0% (−8.8% to 18.8%)
END, *n* (%)	11 (17.5)	29 (20.3)	0.83 (0.39–1.79)	0.99 (0.42–2.32)	1.19 (0.51–2.81)	2.5% (−9.9% to 15.0%)
Any ICH, *n* (%)	12 (19.4)	15 (10.5)	2.05 (0.90–4.68)	2.30 (0.93–5.69)	2.18 (0.84–5.65)	9.3% (−2.5% to 21.0%)
sICH (by NINDS criteria)^c^, *n* (%)	6 (9.7)	10 (7.0)	1.43 (0.49–4.11)	1.43 (0.49–4.11)^*^	1.67 (0.51–5.46)	3.9% (−5.4% to 13.2%)
sICH (by ECASS-II criteria)^d^, *n* (%)	4 (6.5)	7 (4.9)	1.34 (0.38–4.75)	1.34 (0.38–4.75)^*^	1.65 (0.40–6.86)	2.6% (−5.1% to 10.3%)
sICH (by ECASS-III criteria)^e^, *n* (%)	3 (4.8)	4 (2.8)	1.77 (0.38–8.14)	1.77 (0.38–8.14)^*^	2.48 (0.40–15.32)	3.0% (−3.4% to 9.4%)
sICH (by SITS-MOST criteria)^f^, *n* (%)	3 (4.8)	1 (0.7)	7.22 (0.74–70.83)	7.22 (0.74–70.83)^*^	5.02 (0.51–49.44)	4.1% (−2.0% to 10.2%)
All-cause mortality at 90 days, *n* (%)	6 (9.5)	9 (6.3)	1.57 (0.53–4.61)	1.57 (0.53–4.61)^*^	1.63 (0.45–5.89)	3.1% (−5.4% to 11.6%)

PSM, propensity score matching; OR, odds ratio; CI, confidence interval; RD, absolute rate difference; mRS, modified Rankin scale; ENI, early major neurologic improvement; END, early neurologic deterioration; ICH, intracranial hemorrhage; sICH, symptomatic intracranial hemorrhage; NINDS, National Institute of Neurological Diseases and Stroke; ECASS-II, European Cooperative Acute Stroke Study II; ECASS-III, European Cooperative Acute Stroke Study III; SITS-MOST, Safe Implementation of Thrombolysis in Stroke Monitoring Study. One patient receiving standard-dose alteplase died before follow-up imaging.

* SICH and mortality were not further adjusted with covariates due to their rarity.

a Adjusted for age, sex, NIHSS, onset-to-needle time, perfusion-lesion volume, and posterior circulation stroke using the binary logistic regression models.

b Adjusted for age, NIHSS, confirmed extended time window, perfusion-lesion volume, posterior circulation stroke, onset-to-needle time, large vessel occlusion, and mechanical thrombectomy using PSM.

c Any intracranial hemorrhage with neurologic deterioration (an increase of ≥1 in the NIHSS score) from baseline or death.

d Any intracranial hemorrhage with neurologic deterioration (an increase of ≥4 in the NIHSS score) from baseline or death.

e Any intracranial hemorrhage identified as a predominant cause of neurologic deterioration (an increase of ≥4 in the NIHSS score) from baseline or death.

f Large local or remote parenchymal hematoma type 2 combined with neurologic deterioration (an increase of ≥4 in the NIHSS score) from baseline or death.

**Figure 2:**
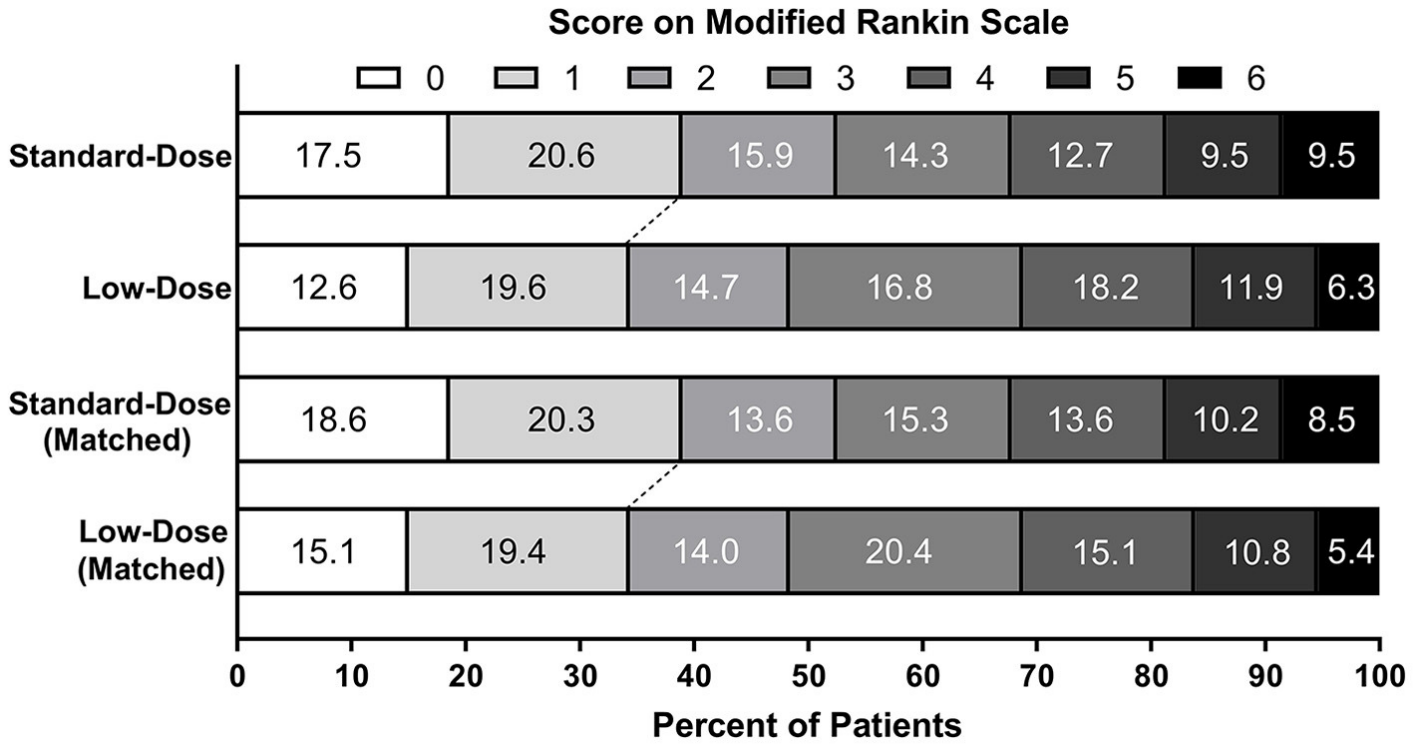
Distribution of scores on the modified Rankin Scale at 90 days before and after PSM.

In the PSM cohort, the proportions of patients achieving excellent functional recovery were 38.9% in the standard-dose group and 34.5% in the low-dose group [adjusted OR = 1.22, 95% CI 0.62–2.39; adjusted RD = 4.6%, 95% CI (−11.2 to 20.3%); Figure 2]. There were no statistical differences observed regarding the incidences of functional independence, ENI, END, any ICH, sICH, and 90-day mortality between the two groups. The point estimates of the primary and secondary outcomes were analogous to those in the original cohort, except for END (adjusted OR = 1.19, 95% CI 0.51–2.81). The sensitivity analysis using the multivariate logistic regression models showed similar results after adjusting for potential confounders, including age, sex, NIHSS, onset-to-needle time, perfusion-lesion volume, and posterior circulation stroke.

### Subgroup analysis

**Figure 3:**
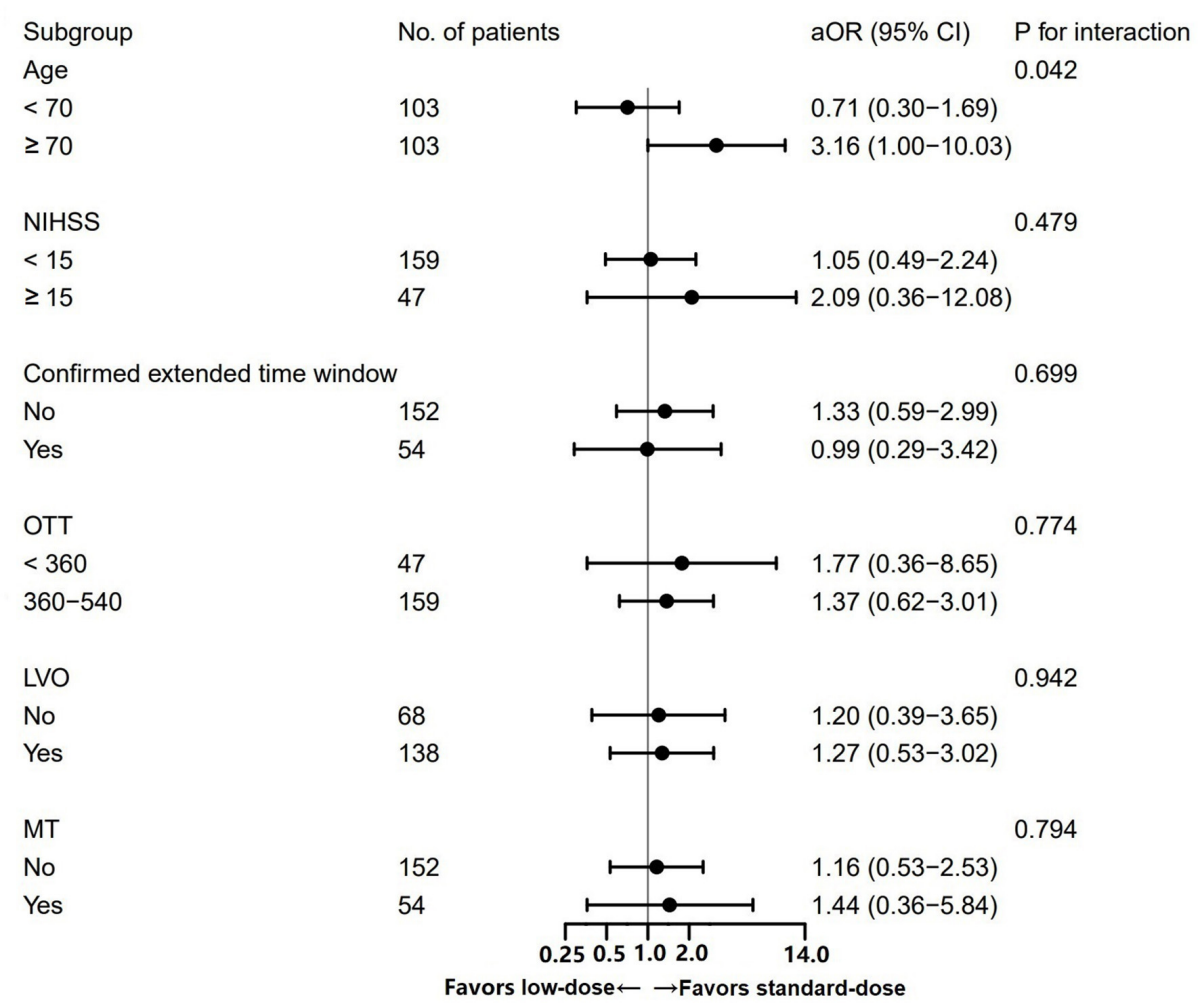
Subgroup analysis of the excellent functional recovery between patients treated with standard- vs. low-dose alteplase.

A significant interaction between the alteplase dose and age was observed (*P*_*interaction*_ = 0.042; Figure 3). Among patients aged ≥70 years, the administration of a standard dose was associated with the higher odds of excellent functional recovery compared to a low dose (OR = 3.16, 95% CI 1.00–10.03). Among patients <70 years, the opposite result was obtained (OR = 0.71, 95% CI 0.30–1.69). No significant heterogeneity in the effect of alteplase dose in the case of mRS scores of 0–1 was observed across other subgroups.

## Discussion

Our real-world clinical cohort study suggested that compared to standard-dose alteplase, low-dose alteplase might exhibit similar effectiveness in AIS patients aged <70 years but not in those aged ≥70 years, with favorable perfusion-imaging profiles in the unknown or extended time window. Moreover, low-dose alteplase was not associated with a decreased rate of sICH.

To date, two imaging algorithms, including the perfusion-core mismatch and diffusion-weighted imaging-fluid-attenuated inversion recovery (DWI-FLAIR) mismatch, have been demonstrated to be effective and safe in guiding IVT beyond the traditional 4.5-h time window from the last known well time (4, 23). The Thrombolysis for Acute Wake-Up and Unclear-Onset Strokes with Alteplase at 0.6 mg/kg (THAWS) trial is the only RCT to investigate the effect of low-dose alteplase in the unknown time window (15). Although the trial did not demonstrate significant benefits with respect to favorable outcomes, it did confirm the safety of administering low-dose alteplase to AIS patients with a DWI-FLAIR mismatch on MRI as compared to antithrombotic treatment (15). Notably, the subgroup of patients with moderate ischemic-lesion volume (ASPECTS 5-8) on DWI and no corresponding hyperintensity on FLAIR exhibited greater benefits from low-dose IVT with regard to favorable outcomes than antithrombotic treatment in the subsequent sub-study (24). Building upon the concept of salvageable penumbra, the present study further compared the true effect of different doses of intravenous alteplase, guided by perfusion-core mismatch on CT perfusion imaging, in the unknown or extended time window.

The patients enrolled in our study shared several similarities with those in the alteplase group of the EXTEND trial, including stroke severity, ischemic-core volume, median ONT time, and proportions of LVO and confirmed extended time window. The differences lie in that our patients had lower age, shorter DNT time, and a higher proportion of large artery atherosclerosis. Despite these differences, the rates of excellent functional recovery were comparable (38.1 vs. 32.2%) between the two groups in our study, which were similar to 35.4% in the alteplase group of the EXTEND trial (4). Furthermore, the early responses to alteplase (including ENI and END) did not differ between the two groups. Similar findings were observed in a multicenter study in Taiwan, which investigated the effect of different doses of alteplase administered within 3 to 4.5 h of symptom onset, and in a study comparing the effects of different doses of alteplase in bridging therapy in Vietnam (25, 26). Nevertheless, the non-significant differences between standard- and low-dose alteplase might be due to the true therapeutic effects or false-negative error caused by the limited sample size and confounding bias of the observational study.

In subgroup analysis, we found that patients aged ≥70 years derived greater benefits from standard-dose alteplase than from low-dose alteplase with respect to excellent functional recovery. Consistent with our finding, the INtravenous Thrombolysis REgistry for Chinese Ischaemic Stroke (INTRECIS) registry showed a trend favoring the standard dose among patients aged ≥65 years (OR = 1.04 for excellent functional outcome, 95% CI 0.57–1.87) compared to those aged <65 years (OR = 2.69, 95% CI 1.22–6.75), although no significant interaction between the alteplase dose and age (<65 vs. ≥65 years, *P*_*interaction*_ = 0.06) was observed (27). In addition, a study using data from the ENCHANTED trial found that younger patients with favorable baseline characteristics, such as lower SBP, mild neurologic deficits, and no comorbidities, were more likely to benefit from low-dose alteplase (28). However, the significant heterogeneity of age might be caused by a small sample size of patients aged ≥70 years. Therefore, more studies on the relationships between the effectiveness of different doses of alteplase and age are still needed.

Regarding the safety profile, our study found low rates of sICH following IVT, with 4.8 and 0.7% (SITS-MOST criteria) observed in the standard- and low-dose groups. These rates were comparable to those in patients treated with different doses of alteplase within 4.5 h in the ENCHANTED trial (2.1% and 1.0%) (11), patients treated with low-dose alteplase in the unknown time window in the THAWS trial (1.4%) (15), and patients treated with standard-dose alteplase in the unknown or extended time window up to 9 h in the EXTEND trial (6.2%) (4). In our study, treating physicians were more likely to administer low-dose alteplase to older patients, considering the high risk of bleeding. However, we did not find an association between low-dose alteplase and the significantly decreased risk of sICH, which was different from the results of the ENCHANTED trial. This finding was consistent with the results of previous real-world studies conducted in China (25, 27). Our study results indicate that standard-dose alteplase may not lead to significantly higher rates of sICH than low-dose alteplase in the unknown or extended time window. The overall low rates of sICH might be partially attributed to the small ischemic-core volume detected upon admission (median volume 4 ml). Previous studies have shown that a larger ischemic core volume increases the risk of hemorrhage after IVT and decreases the likelihood of functional independence (29, 30). A retrospective cohort study in Germany demonstrated that IVT with alteplase in the extended time window with no upper time limit still appeared safe, and the median core volume was only 0 ml, as assessed by the RAPID software (31).

Our study enrolled 35 (17.0%) patients with PCS, which is in line with the previous findings indicating that PCS accounts for 12–19% of all IVT-treated strokes (32). Despite the lack of RCT data on the efficacy and safety of IVT in PCS, a recent meta-analysis of 10,303 AIS patients from real-world studies demonstrated that IVT in PCS was associated with a 50% reduction in the risk of sICH and comparable functional outcomes to IVT in ACS within 4.5 h of stroke onset (33). Furthermore, time to IVT in PCS tended not to critically influence the risk of ICH and chances for unfavorable outcomes as it did in ACS (34). Macha et al. investigated the effect of IVT on PCS in the unknown or extended time window using perfusion imaging (algorithm: mismatch ratio >1.4) (35). The results showed no differences in the safety and effectiveness between IVT in PCS and ACS, with a trend toward less ICH in PCS. Therefore, IVT in PCS beyond 4.5 h seemed feasible and safe in the real-world clinical setting. Given that the currently available automated perfusion software is designed for ACS and is insensitive to PCS, future research is needed with respect to the development of automated perfusion software to specifically detect ischemia in the posterior circulation.

The automated perfusion software utilized in our study was MIStar, which is different from RAPID used in the EXTEND trial. Both kinds of software differ in algorithms of deconvolution, and one particular difference is that RAPID does not theoretically correct the delay and dispersion in contrast traveling from the proximal arteries to the ischemic area. Consequently, the ischemic core and penumbra volumes might be overestimated (36, 37). A recent *post-hoc* analysis of the EXTEND trial reprocessed the perfusion imaging with MIStar and demonstrated that patients selected by MIStar who met the same target mismatch criteria as per the original trial could still benefit from IVT, with even better functional recovery (mRS 0–1, adjusted OR = 2.23, 95% CI: 1.08–4.58) compared with RAPID-selected patients in the original trial (mRS 0–1, adjusted OR = 1.88) (37). Therefore, the use of MIStar would not reduce the overall benefit of alteplase among AIS patients with target mismatch in the unknown or extended time window.

In reality, the disease burden of stroke in China is the most severe across the world. According to a national cross-sectional report, the rates of IVT have improved a lot from <3% before 2013 to 5.64% between 2019 and 2020 (2). Nonetheless, there is still a giant gap between China and other developed countries. The major reasons for not performing IVT are likely due to delayed admission, high prices of thrombolytic drugs, and concerns related to bleeding risk (38). To the best of our knowledge, this study was the first to demonstrate that low-dose alteplase at 0.6 mg/kg seemed to have a similar effect to standard-dose alteplase at 0.9 mg/kg for AIS in the unknown or extended time window. Taking into account both effectiveness and safety, low-dose alteplase might be used as an alternative treatment option for Chinese AIS patients aged <70 years with favorable perfusion-imaging profiles outside the 4.5-h time window in clinical practice.

The present study had several limitations. First, this is a non-randomized, single-center retrospective study. The choice between standard- and low-dose alteplase was determined by the treating physicians, and the final decisions might be influenced by various clinical factors. Therefore, the selection bias could not be avoided. Although different statistical methods were used to account for baseline imbalances, there might exist potential and unknown confounders that were not included in our study. Second, owing to a small sample size, our study was likely underpowered to detect differences in treatment effects, and drawing any definite conclusions were impossible. Third, we did not perform an analysis of the imaging outcomes (e.g., rates of reperfusion and the final infarct volume) between the two groups due to a lack of follow-up MRI post-IVT in some patients. Finally, patients receiving MT were also included in this study. Although the proportions of these patients were balanced between the two groups before and after PSM, this might potentially affect the final treatment effects.

## Conclusion

This observational study indicated that perfusion imaging-guided IVT with standard-dose alteplase might be associated with comparable or superior functional outcomes compared to low-dose alteplase in Chinese AIS patients aged <70 years and ≥70 years in the extended or unknown time window, without significantly increasing the risk of sICH. For patients younger than 70 years, low-dose alteplase might be a feasible alternative, but further validation through future RCTs and prospective registries is necessary.

## Data availability statement

The original contributions presented in the study are included in the article/Supplementary material, further inquiries can be directed to the corresponding author.

## Ethics statement

The studies involving human participants were reviewed and approved by Research Ethics Committee of the First Affiliated Hospital of Soochow University (No. 2022029). Written informed consent for participation was not required for this study in accordance with the national legislation and the institutional requirements.

## Author contributions

ZW: study design, data collection and analysis, and manuscript writing. KJ: data collection and analysis. QF: manuscript revision. All authors have read and approved the final version of the manuscript.
