# Supplementary material for: Low-dose vs. standard-dose intravenous alteplase for acute ischemic stroke with unknown time of onset
Source: Front Neurol. 2023 Apr 28;14:1165237. doi: 10.3389/fneur.2023.1165237 (PMC10175638; doi:10.3389/fneur.2023.1165237)
Supplement: Supplementary file 1 [file Image_1.pdf]

Supplementary Material

Low-dose vs. Standard-dose Intravenous Alteplase for Acute Ischemic Stroke with Unknown Time of Onset

Zekun Wang, Kangxiang Ji and Qi Fang*

*** Correspondence:** Fang Qi: fangqi_008@126.com

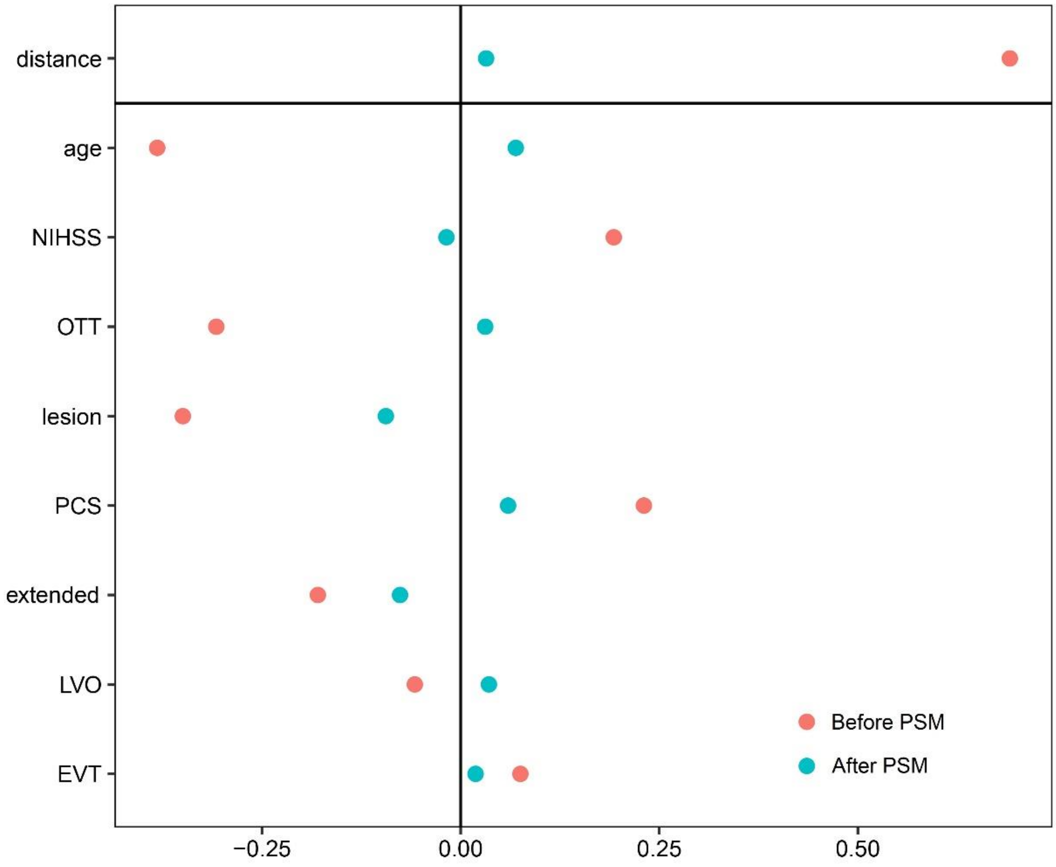

Supplementary Figure 1 Standardized mean difference in variables included in the propensity score before and after matching in the patient cohort
